# Biphasic Outbreak of Invasive Group A *Streptococcus* Disease in Eldercare Facility, New Zealand

**DOI:** 10.3201/eid2605.190131

**Published:** 2020-05

**Authors:** Kate A. Worthing, Anja Werno, Ramon Pink, Liam McIntyre, Glen P. Carter, Deborah A. Williamson, Mark R. Davies

**Affiliations:** The University of Melbourne at the Peter Doherty Institute for Infection and Immunity, Melbourne, Victoria, Australia (K.A. Worthing, L. McIntyre, G.P. Carter, D.A. Williamson, M.R. Davies);; Canterbury Health Laboratories, South Island, New Zealand (A. Werno);; Canterbury District Health Board, South Island (R. Pink)

**Keywords:** group A Streptococcus, Streptococcus pyogenes, invasive disease outbreak, genomic epidemiology, integrative conjugative element, macrolide resistance, outbreak management, bacteria, New Zealand, antimicrobial resistance, GAS, streptococci

## Abstract

A 3-month outbreak of invasive group A *Streptococcus* disease at an eldercare facility, in which 5 persons died, was biphasic. Although targeted chemoprophylaxis contained the initial outbreak, a second phase of the outbreak occurred after infection control processes ended. To retrospectively investigate the genomic epidemiology of the biphasic outbreak, we used whole-genome sequencing and multiple bioinformatics approaches. Analysis of isolates from the outbreak and isolates prospectively collected during the outbreak response indicated a single S*. pyogenes* *emm*81 clone among residents and staff members. Outbreak isolates differed from nonoutbreak *emm*81 isolates by harboring an integrative conjugative genomic element that contained the macrolide resistance determinant *erm*(TR). This study shows how retrospective high-resolution genomic investigations identified rapid spread of a closed-facilty clonal outbreak that was controlled, but not readily cleared, by infection control management procedures.

*Streptococcus pyogenes*, or group A *Streptococcus* (GAS), is a gram-positive, human-adapted opportunistic bacterial pathogen. GAS causes a wide variety of clinical manifestations, from relatively benign self-limiting infections of the nasopharynx or skin to life-threatening invasive infections such as bacteremia, pneumonia, and necrotizing fasciitis (*1*). The incidence of invasive GAS infections is highest among older adults (*2*,*3*), particularly those living in long-term care facilities (*2*,*4*). Outbreaks of GAS infections are often linked with distinct epidemiologic markers such as *emm* type (*5*,*6*). *emm* typing is a sequence-based method that analyzes heterogeneity in the 5′ end of the ubiquitous *emm* gene that encodes the M-protein (*7*). Although *emm* typing provides useful information about the potential relatedness of outbreak isolates, whole-genome sequencing enables outbreak investigations to proceed with a far greater level of discrimination than single-gene typing of GAS isolates (*8*,*9*). For this study, we used multiple whole-genome–based approaches to examine the genetic relationships and molecular drivers of a biphasic GAS outbreak in an eldercare facility in which 14 persons became ill and 5 died.

## Methods

### Setting

During winter 2014, an outbreak of invasive and noninvasive GAS disease occurred in an eldercare facility in South Island, New Zealand. The outbreak was recognized by a senior laboratory scientist who noted the sudden increase in positive blood cultures from the facility. The outbreak occurred in 2 phases. The first phase started in late May 2014 and ended when the last case-patient (a resident) was hospitalized in early June 2014. The initial 3 case-patients were admitted within 24 hours of each other, and GAS was isolated from blood or tissue cultures from all 3 case-patients. During the first phase, 6 case-patients with confirmed GAS infection were hospitalized; 5 died of presumed sepsis. Outbreak investigations and control measures were subsequently implemented and included screening staff members and residents by collecting throat swab samples and providing targeted chemoprophylaxis for residents and staff members who were in direct contact with case-patients. 

These interventions continued until early July 2014; however, in late July, the outbreak recurred and continued until mid August 2014. In this second phase, a staff member was hospitalized with severe pharyngitis, after which 5 residents were hospitalized with soft tissue infections, septicemia, or both.

### Case Definitions

According to disk-diffusion testing, all GAS isolates collected during the first phase were susceptible to penicillin but resistant to erythromycin. Accordingly, a suspected case-patient was defined as any resident or staff member from the facility who was unwell from early June 2014 through mid-November 2014, and a confirmed case-patient was any person from the facility from whom erythromycin-resistant GAS was cultured from blood, throat, or skin samples. The definition of a suspected case-patient was kept intentionally broad because of the wide-ranging symptoms among initial case-patients. A carrier was defined as any asymptomatic person from the facility from whom erythromycin-resistant GAS was isolated from a skin or throat swab sample.

### Comparison of Outbreak Isolates with Nonoutbreak Isolates

After the initial 3 cases were confirmed, throat swab samples were collected from all residents and from facility staff members who worked in nursing, kitchen, or waste collection. Swab samples were also collected from any skin lesion on staff members or residents. Hospital staff working in the wards where residents had been admitted were also asked to consent to collection of throat swab samples. The outbreak isolates were compared with nonoutbreak isolates for contextual purposes. Nonoutbreak isolates were defined as clinical isolates submitted to the Institute of Environmental Science and Research (ESR) in New Zealand from across New Zealand during 2002–2014. Although including only contemporaneous nonoutbreak isolates would have been ideal (that is, only those collected in 2014), New Zealand’s small population and the fact that GAS infections are not notifiable in New Zealand meant that we had to select nonoutbreak isolates over a broader time frame. All outbreak and nonoutbreak isolates underwent initial *emm* typing at ESR according to previously described methods (*7*). We included in our compariative analyis only nonoutbreak isolates that had the same *emm* type as the outbreak isolates. Data collection was approved by the Medicine, Dentistry and Health Sciences Human Ethics Sub-Committee at the University of Melbourne (ID no. 1853078).

### Genome Sequencing and Assembly

We performed genome sequencing and assembly for outbreak and nonoutbreak isolates. All isolates underwent Illumina whole-genome sequencing (https://www.illumina.com). To enable fine-mapping of the outbreak, we completely sequenced a representative *emm*81 outbreak isolate, DMG1800716, by using Pacific Biosciences long-read technology (https://www.pacb.com). To validate the consensus assembly of the reference genome, we used Illumina short reads. We used Prokka (*10*) with manual curation to annotate the final sequence and SPAdes version 3.9.0 (*11*) for de novo assembly of raw Illumina reads into draft assemblies. We performed pairwise BLAST (https://blast.ncbi.nlm.nih.gov/Blast.cgi) comparisons of the 55 *emm*81 genomes relative to DMG1800716 by using the BLAST Ring Image Generator (*12*). We submitted the complete genome sequence of DMG1800716 to GenBank under accession no. CP027771. Short reads of all sequenced isolates are available at the National Center for Biotechnology Information sequence read archive (https://www.ncbi.nlm.nih.gov/sra) under BioProject PRJNA494270.

### Phylogenetic Analyses

To determine whether outbreak isolates were genetically related, we mapped the genomes of the outbreak isolates and nonoutbreak isolates from across New Zealand to the newly generated 1,869,673-bp *emm*81 outbreak reference genome, DMG1800716 (Appendix 1). We inferred phylogenetic relationships by both maximum-likelihood and Bayesian assessment of core-genome single-nucleotide polymorphism (SNPs). We used consensus SNP alignments to build a maximum-likelihood tree with RAxML version 8.0.1 (*13*) and assessed temporal phylogenetic analysis by using BEAST version 2.4.7 (*14*), and a Hasegawa-Kishino-Yano plus gamma site model with a strict clock model after assessing temporal signal by using TempEst (*15*). 

## Results

### Clinical Epidemiology of the Outbreak

During the 2 phases of the outbreak, 14 cases of erythromycin-resistant GAS infection were confirmed: 10 in residents and 4 in staff members. Eleven of the confirmed cases (10 in residents, 1 in a staff member) were detected by swabbing of unwell persons with suspected cases, and the other 3 confirmed cases were identified by prospective sampling of all 75 residents and 30 hospital staff members (for each of these staff members, the outbreak strain was isolated from skin lesions on their hands). Prospective swabbing also identified 1 resident as a carrier (erythromycin-resistant GAS was isolated from the resident’s throat).

The average case-patient age was 79.5 years. Residents exhibited a variety of signs and symptoms (e.g., fever, malaise, suspected septic arthritis, diarrhea and vomiting, abdominal pain, and skin lesions). One staff member was hospitalized with severe pharyngitis; the other staff members were treated at home for minor skin infections. 

During the first phase of the outbreak, a characteristic feature was the rapidity with which case-patient conditions deteriorated; 1 died within a few hours of symptom onset. Five confirmed case-patients, all residents, died of streptococcal sepsis during the first phase. 

During the second phase of the outbreak, no deaths were reported. Five persons had suspected cases (4 residents, 1 staff member) during the outbreak but were excluded from this analysis because erythromycin-resistant GAS was not isolated.

### Outbreak Isolates 

During our investigation, we obtained 18 erythromycin-resistant GAS isolates, which were cultured from a variety of body sites including blood, throat, and soft tissue (Table). For 2 residents, identical outbreak strains were isolated from 2 different body sites; the remaining isolates each came from different patients. The phenotypic antimicrobial sensitivity pattern of the outbreak isolates included susceptibility to penicillin, methicillin (oxacillin), amoxicillin, and vancomycin and resistance to erythromycin with inducible resistance to clindamycin. No GAS with this antibiogram was cultured from the 65 screening throat swab samples from external hospital staff (those working in wards where case-patients were admitted).

**Table:** Details of *Streptococcus pyogenes emm*81 strains in study of biphasic outbreak of invasive group A *Streptococcus* disease in eldercare facility, New Zealand*

Isolate name	Sample date	Specimen type	Source	Region	SRA biosample accession no.
DMG1800704	2014 May	Hip joint†	Outbreak, resident	South Island	SAMN10160123
DMG1800705	2014 May	Blood†	Outbreak, resident	South Island	SAMN10160124
DMG1800706	2014 May	Blood	Outbreak, resident	South Island	SAMN10160125
DMG1800707	2014 May	Blood	Outbreak, resident	South Island	SAMN10160126
DMG1800708	2014 Jun	Throat	Outbreak, resident	South Island	SAMN10160127
DMG1800709	2014 Jun	Throat‡	Outbreak, resident	South Island	SAMN10160128
DMG1800710	2014 Jun	Leg ulcer‡	Outbreak, resident	South Island	SAMN10160129
DMG1800711	2014 Jun	Leg lesion	Outbreak, resident	South Island	SAMN10160130
DMG1800712	2014 Jun	Elbow lesion	Outbreak, resident	South Island	SAMN10160131
DMG1800713	2014 Jun	Fingernail	Outbreak, staff	South Island	SAMN10160132
DMG1800714	2014 Jun	Hand	Outbreak, staff	South Island	SAMN10160133
DMG1800715	2014 Jul	Throat	Outbreak, staff	South Island	SAMN10160134
DMG1800716§	2014 Aug	Blood	Outbreak, resident	South Island	SAMN10160135
DMG1800717	2014 Aug	Leg wound	Outbreak, resident	South Island	SAMN10160136
DMG1800718	2014 Aug	Foot ulcer	Outbreak, resident	South Island	SAMN10160137
DMG1800719	2014 Aug	Finger	Outbreak, staff	South Island	SAMN10160138
DMG1800720	2014 Aug	Blood	Outbreak, resident	South Island	SAMN10160139
DMG2000217	2014 Aug	Wound	Outbreak, resident	South Island	SAMN14177818
DMG1800721	2012 Jan	Blood	Nonoutbreak	Not recorded	SAMN10160140
DMG1800722	2012 Jan	Throat	Nonoutbreak	Not recorded	SAMN10160141
DMG1800723	2013 Jan	Throat	Nonoutbreak	Not recorded	SAMN10160142
DMG1800724	2013 Jan	Blood	Nonoutbreak	Not recorded	SAMN10160143
DMG1800725	2014 Jan	Blood	Nonoutbreak	Not recorded	SAMN10160144
DMG1800726	2002 Jan	Blood	Nonoutbreak	South Island	SAMN10160145
DMG1800727	2003 Jan	Blood	Nonoutbreak	North Island	SAMN10160146
DMG1800728	2003 Jan	Blood	Nonoutbreak	North Island	SAMN10160147
DMG1800729	2003 Jan	Blood	Nonoutbreak	North Island	SAMN10160148
DMG1800730	2005 Jan	Blood	Nonoutbreak	South Island	SAMN10160149
DMG1800731	2005 Aug	Blood	Nonoutbreak	North Island	SAMN10160150
DMG1800732	2006 Jan	Blood	Nonoutbreak	North Island	SAMN10160151
DMG1800733	2006 Sep	Blood	Nonoutbreak	South Island	SAMN10160152
DMG1800734	2006 Sep	Blood	Nonoutbreak	North Island	SAMN10160153
DMG1800735	2007 Jun	Blood	Nonoutbreak	South Island	SAMN10160154
DMG1800736	2007 Jun	Blood	Nonoutbreak	South Island	SAMN10160155
DMG1800737	2007 Jun	Blood	Nonoutbreak	North Island	SAMN10160156
DMG1800738	2008 May	Blood	Nonoutbreak	South Island	SAMN10160157
DMG1800739	2010 May	Blood	Nonoutbreak	North Island	SAMN10160158
DMG1800740	2010 May	Blood	Nonoutbreak	North Island	SAMN10160159
DMG1800741	2010 May	Blood	Nonoutbreak	North Island	SAMN10160160
DMG1800742	2001 Feb	Blood	Nonoutbreak	North Island	SAMN10160161
DMG1800743	2011 Feb	Blood	Nonoutbreak	North Island	SAMN10160162
DMG1800744	2011 Mar	Blood	Nonoutbreak	North Island	SAMN10160163
DMG1800745	2012 May	Blood	Nonoutbreak	North Island	SAMN10160164
DMG1800746	2012 May	Blood	Nonoutbreak	North Island	SAMN10160165
DMG1800747	2012 Jun	Blood	Nonoutbreak	North Island	SAMN10160166
DMG1800748	2013 Jan	Blood	Nonoutbreak	North Island	SAMN10160167
DMG1800749	2013 Feb	Blood	Nonoutbreak	North Island	SAMN10160168
DMG1800750	2013 Mar	Blood	Nonoutbreak	North Island	SAMN10160169
DMG1800751	2013 Jul	Throat	Nonoutbreak	North Island	SAMN10160170
DMG1800752	2013 Dec	Blood	Nonoutbreak	North Island	SAMN10160171
DMG1800753	2014 May	Throat	Nonoutbreak	North Island	SAMN10160172
DMG1800754	2014 Jun	Blood	Nonoutbreak	North Island	SAMN10160173
DMG1800755	2014 Jul	Aspirate	Nonoutbreak	South Island	SAMN10160174
DMG1800756	2014 Sep	Blood	Nonoutbreak	North Island	SAMN10160175
DMG1800757	2014 Nov	Blood	Nonoutbreak	North Island	SAMN10160176

*SRA, National Center for Biotechnology Information sequence read archive (https://www.ncbi.nlm.nih.gov/sra). †Isolates with this symbol are from the same resident. ‡Isolates with this symbol are from the same resident. §Reference genome.

### Outbreak Management Interventions

After the initial 3 cases were confirmed and the outbreak was recognized, public health staff members initiated targeted chemoprophylaxis. A 10-day course of penicillin or amoxicillin was given to all staff members, any resident who was unwell or had been in contact with a case-patient, and any resident from whom GAS was isolated. The 4 staff members with outbreak strain infections stayed away from work until they had completed their course of antimicrobial therapy, their clinical signs of infections had resolved, and a throat swab sample culture was negative. Outbreak control measures initially continued for 1 month after the last case in the first outbreak phase was identified.

When the outbreak recurred, additional surveillance and environmental control measures were initiated and continued for 3 months after the last case of the second phase was identified. Other additional control measures included educating staff and residents about hand hygiene, monitoring the temperature of any resident with a skin lesion, cleaning all furniture and upholstery with diluted bleach where possible, replacing all toothbrushes, using disposable wound dressing trays rather than trolleys, inspecting the hands of staff members for skin lesions daily, and instructing the hospital to collect blood and throat swab samples for culture from any residents admitted from this eldercare facility and to place them in a single room. Items used communally by residents and staff (e.g., salt and pepper shakers, portable telephones) were cleaned with diluted bleach after meals or each use.

### Genomic Epidemiology

Molecular analysis of the 18 outbreak GAS strains indicated that they all contained the *emm*81.0 gene allele. Only 5 contemporaneous nonoutbreak *emm*81 isolates were collected in New Zealand during 2014; the remaining 32 nonoutbreak isolates were collected during 2002–2013. Core-genome comparisons of the 18 outbreak strains with the 37 nonoutbreak *emm*81 isolates showed that the outbreak isolates were highly clonal and formed a separate clade in the *emm*81 phylogeny (Figure, panel A). Although 336 core-genome SNPs were identified among all *emm*81 isolates studied, no SNPs were identified between 15 of the outbreak isolates and 1 SNP difference was identified in the remaining 3 isolates. One isolate from a staff member differed from the outbreak isolates by a single SNP in the *murM* locus; the remaining isolates from staff members were indistinguishable from isolates from residents (Appendix 2). These data suggest spread of the outbreak clone between staff members and residents to which directionality cannot be inferred. A single nonoutbreak isolate, DMG1800755, differed from the outbreak clade by 2 SNPs. The isolate came from an aspirate from a patient in the same southern region of New Zealand in mid-2014, around the temporal midpoint of the outbreak. The remaining nonoutbreak isolates showed a distant evolutionary relationship to the outbreak *emm*81 lineage.

**Figure:**
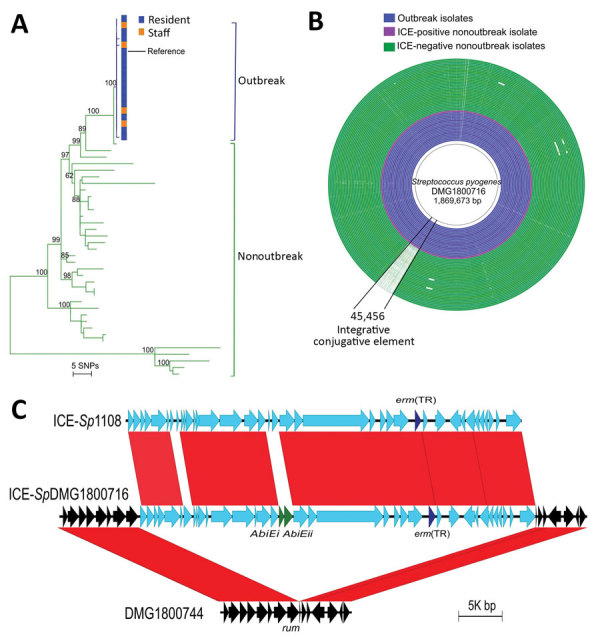
Comparative genomic analyses of 55 (18 outbreak and 37 nonoutbreak) associated *emm*81 group A *Streptococcus* (GAS) isolates from New Zealand, 2014. A) Midpoint-rooted maximum-likelihood phylogenetic analysis of the *emm*81 GAS population based on alignment of 336 high-quality single-nucleotide polymorphisms. Green branches indicate nonoutbreak isolates and blue branches indicate the clonal outbreak isolates. Outbreak isolates obtained from eldercare residents (blue) and staff members (orange) were indistinguishable at the whole-genome level. Numbers on major internal nodes indicate branch support as a percentage over 100 bootstrap replicates. The tree was created by using RAxML (*13*) and annotated by using iTOL (*16*). B) Comparative analyses of 55 *emm*81 draft genome assemblies from outbreak (blue) and nonoutbreak (green) isolates mapped against a new reference GAS genome from the outbreak, DMG1800716. A large DNA sequence coinciding with a 45.4-kb ICE, ICE-*Sp*DMG1800716, is absent in the nonoutbreak isolates compared with all outbreak isolates. The image was created by using BLAST Ring Image Generator (*12*). C) Schematic representation and pairwise sequence comparison (BLASTn, https://blast.ncbi.nlm.nih.gov) of ICE-*Sp*DMG1800716 relative to the closest known homologue, ICE-*Sp*1108 (*17*). The genomic integration site of ICE-*Sp*DMG1800716 is shown relative to a nonoutbreak *emm*81 isolate, DMG1800744. Red bars refer to 100% BLASTn homology as determined by Easyfig (*18*). The macrolide resistance gene *erm*(TR) is shown in dark blue and the abortive infection genes (AbiE) in green. ICE, integrative conjugative element; SNPs, single-nucleotide polymorphisms.

To understand molecular differences between the outbreak *emm*81 lineage and the unrelated nonoutbreak *emm*81 isolates, we investigated genome-wide heterogeneity of the 55 *emm*81 genomes. We screened core genomes for mutations within the key GAS regulatory genes *covR/S*, *ropB*, *mga*, which had previously been linked to increased virulence among GAS isolates (*1*); we found no differential mutations between outbreak and nonoutbreak isolates. Comparison of the accessory (variable) genome content of the 55 *emm*81 isolates revealed that all isolates sampled from South Island during the outbreak period harbored an integrative conjugative element (ICE); all other nonoutbreak isolates sampled during the outbreak period, which were all from other geographic regions, were ICE negative (Figure, panel B). The outbreak ICE element, called ICE-*Sp*DMG1800716, shared 99% nucleotide sequence homology with ICE-*Sp*1108, previously described for an erythromycin-resistant GAS isolate from Italy (*17*) (Figure, panel C). Comparative analysis revealed that ICE-*Sp*DMG1800716 contained the inducible macrolide-resistance gene, *erm*(TR). ICE-*Sp*DMG1800716 also harbored the abortive infection operon, AbiE operon, which is associated with bacteriophage resistance and stabilization of extrachromosomal elements (*17*). The ICE was integrated between the 3′ end of the 23s tRNA methyltransferase (*rum*) gene (*17*) and the 5′ end of a phosphorylase gene of a representative nonoutbreak isolate (DMG1800744) (Figure, panel C). Bayesian temporal analysis of the *emm*81 population indicated that ICE-*Sp*DMG1800716 was acquired during 2007–2013 and that the ICE-positive clade subsequently expanded in 2013 (95% confidence range 2013–2014; Appendix 1 Figure).

## Discussion

Through our clinical and genomic epidemiologic analyses, we determined that a fatal GAS outbreak in an eldercare facility was associated with a single *emm*81 GAS clone that was resistant to erythromycin and exhibited inducible clindamycin resistance. *emm*81 GAS is one of the most common M-types that causes invasive disease in New Zealand (*3*). The role of *emm*81 as a global GAS strain is highlighted by its inclusion in the experimental 30-valent M-protein vaccine (*19*). Traditional typing methods, such as *emm* typing, would not have had the discriminatory power to differentiate the outbreak isolates from other *emm*81 isolates that were already in New Zealand. Along with other recent reports of GAS outbreaks of a single *emm* type (*8*,*9*), our study highlights the utility of whole-genome sequencing as an epidemiologic tool for GAS outbreak investigations.

Comparative analyses of the outbreak clone with 37 nonoutbreak *emm*81 isolates identified that the outbreak clone had acquired a macrolide resistance determinant within a putative integrative and conjugative element, ICE-*Sp*DMG1800716. Outbreaks of GAS disease have previously been linked to the acquisition of mobile genetic elements, such as an ongoing polyclonal *emm*12 and *emm*1 scarlet fever outbreak in Hong Kong and mainland China associated with horizontal acquisition of multidrug resistance and a superantigen-encoding prophage (*6*). Outbreaks of invasive GAS disease have also been associated with acquisition of, or mutations within, genotypic regulatory systems that result in increased phenotypic virulence (*20*,*21*). However, this clonal invasive GAS outbreak differs from previously reported outbreaks (*6**,**20*,*21*) because it was linked primarily to the acquisition of a transposable element with no obvious virulence determinant. Widespread use of macrolides in New Zealand, particularly in elderly patients and during the winter, when this outbreak occurred (*22*), may well have contributed to the selection and expansion of the macrolide-resistant outbreak clone.

In addition to harboring macrolide-resistance genes, the integrative conjugative element in the outbreak isolates also contained the abortive infection protein AbiE, which may have contributed to the relative fitness of the outbreak isolates. AbiE may confer bacteriophage resistance and has been shown to stabilize extrachromosomal elements such as plasmids (*23*); thus, its presence may have helped maintain ICE-*Sp*DMG1800716 within the genomes of the outbreak isolates.

GAS carriage among healthcare workers in this and other outbreaks serves as a reminder that staff member sampling is integral to GAS outbreak investigations (*9*). Such practices, although common in hospital settings, are not universally followed during investigations of outbreaks in long-term care or eldercare facilities (*5*). In addition to a geographic and temporal link between the outbreak isolates and their closest nonoutbreak relative (both being from southern New Zealand and isolated in 2014), no contact history could be determined between the clinical nonoutbreak isolate and the outbreak facility. We therefore hypothesize that the outbreak probably commenced from an unsampled community source that gained entry to the facility by contact with either a resident or staff member. Although an environmental source is unlikely, environmental sampling was not undertaken; thus, fomites such as communal dinnerware or telephones could have been the common source of infection that resulted in the second phase of the outbreak. In a review of 17 reports of GAS outbreaks in long-term care facilities, fomites were not definitively implicated in outbreak transmission (*5*); therefore, an environmental source indeed seems less likely as a source of the recurrence of this outbreak and an unsampled human source seems more likely.

As was the case for other reported outbreaks in long-term care facilities (*8*,*9*), improved infection control measures and chemoprophylaxis were the cornerstones of outbreak control in this outbreak. Although infection control measures are undoubtedly of utmost importance, the evidence as to whether targeted or mass chemoprophylaxis is preferable in eldercare settings is conflicting, because risk for secondary invasive GAS infection is higher among elderly persons than among other contacts (*24*,*25*). Authors of a recent UK study demonstrated a considerably increased risk for invasive GAS infection among household contacts, particularly for persons >75 years of age, for whom the fatality rate for secondary cases was 19% (*24*). They suggested that, even in nonoutbreak settings, targeted chemoprophylaxis for elderly household contacts of invasive GAS patients should be considered (*24*). It is conceivable that the targeted chemoprophylaxis undertaken during the first phase of this outbreak prevented some cases, yet the occurrence of the second phase suggests that this approach alone was not sufficient. A 2007 review of 17 GAS outbreaks in long-term care facilities similarly found that in 3 facilities, targeted chemoprophylaxis was insufficient for achieving outbreak control and that control was achieved only after the facilities initiated mass chemoprophylaxis to augment existing infection control measures (*5*). More recently, mass chemoprophylaxis was insufficient for halting a multiphase outbreak in 2 long-term care facilities in the United States (*9*). In that study, mass chemoprophylaxis was initiated for all residents and consenting staff; prophylactic coverage was wider than that in our study. Nevertheless, mass chemoprophylaxis in the US outbreak was still only partially effective, and outbreak persistence was attributed mostly to continued lapses in infection control practices. During a GAS outbreak in another long-term care facility, breaches in infection control practices were also noted; prospective assessment of staff members’ wound care and hand hygiene practices found several lapses in each (*26*). In our study, improved infection control practices were initiated, but direct observation of staff undertaking wound care and hand hygiene may have further helped to identify exactly where lapses might have been occurring.

In summary, our data further highlight the potential for invasive GAS to cause rapid and fatal outbreaks, particularly in closed communities such as eldercare facilities. Invasive GAS disease is not notifiable in New Zealand, nor is there mandatory surveillance for invasive GAS infections. The incidence of invasive GAS infections in New Zealand and elsewhere is particularly high among those >75 years of age (*2*,*3*). Our findings add to the growing body of evidence emphasizing the need for improved surveillance and response to invasive GAS infections in at-risk populations, particularly in countries such as New Zealand where active surveillance is not conducted.

Appendix 1 Additional methods and results for study of biphasic outbreak of invasive group A *Streptococcus* disease in nursing care facility, New Zealand.

Appendix 2 Additional results for study of biphasic outbreak of invasive group A *Streptococcus* disease in nursing care facility, New Zealand.
