# Supplementary material for: Biphasic Outbreak of Invasive Group A Streptococcus Disease in Eldercare Facility, New Zealand
Source: Emerg Infect Dis. 2020 May;26(5):841–8. doi: 10.3201/eid2605.190131 (PMC7181915; doi:10.3201/eid2605.190131)
Supplement: Appendix 1 — Additional methods and results for study of biphasic outbreak of invasive group A Streptococcus disease in nursing care facility, New Zealand. [file 19-0131-Techapp-s1.pdf]

Article DOI: https://doi.org/10.3201/eid2605.190131

Biphasic Outbreak of Invasive Group A *Streptococcus* Disease in Eldercare Facility, New Zealand

Appendix 1

Detailed methods

Genome sequencing

All isolates underwent short-read Illumina sequencing while only the reference isolate, DMG1800716 underwent long-read PacBio sequencing. Paired-end multiplex libraries were created and sequenced using the Illumina NextSeq 500 platform or Illumina Hiseq 2500 with a read length of 250 or 100 base pairs respectively. DMG1800716 genomic DNA was extracted using GenElute Bacterial Genomic DNA Extraction kit (Sigma) with a modified protocol that included 50 ug/ml lysozyme and 150 Units/ml mutanolysin. A 20kb library was size selected using BluePippin (Sage Science) and sequenced on the PacBio RSII using the DNA sequencing kit 4.0 v2, C4 chemistry and P6 generation polymerase.

The complete genome assembly of GAS DMG1800716 was determined from a single SMRT cell using the SMRTpipe v2.1.0 with HGAP.2 and Quiver for post-assembly consensus validation. Illumina short reads were used to validate the consensus assembly. The final sequence was annotated using Prokka (*1*) with manual curation. Raw Illumina reads were *de novo* assembled into draft assemblies using SPAdes v.3.9.0 (*2*). Pairwise blast comparisons of the 55 *emm*81 genomes relative to DMG1800716 were performed using the BLAST Ring Image Generator (BRIG) (*3*). The complete genome sequence of DMG1800716 was submitted to GenBank under the accession number CP027771. Short reads of all sequenced isolates are available at the NCBI short read archive (SRA) under the accession numbers provided in Table 1.

Phylogenetic analysis

Phylogenetic relationships were inferred by both maximum-likelihood and Bayesian assessment of single nucleotide polymorphism (SNPs). Illumina paired-end short reads were mapped to the reference sequence (DMG1800716) using BWA-MEM v.0.7.15 with an average coverage of 105x. A total of 1,142 SNPs were identified from the alignment of 55 *emm*81 genomes to DMG1800716 using FreeBayes v.1.0.2. Low complexity regions in the reference genome (DMG1800716) were identified as described previously (*4*). Mobile genetic elements and genomic regions of irregular SNP density were identified in the DMG1800716 reference genome and from the 55 *emm*81 core genome alignment using PHASTER and Gubbins v.2.2.0 respectively (*5*,*6*). All low complexity mapping regions, high SNP density regions and mobile genetic elements were then excised from the alignment resulting in a 1,715,206 bp core genome alignment with a total of 336 vertically acquired SNPs. Consensus SNP alignments were used to build a maximum-likelihood tree with RAxML v8.0.1 (*7*). A general time-reversible model with gamma correction was used in RaxML, performed with 100 bootstrap random resamplings to assess tree support. Temporal phylogenetic analysis was assessed with BEAST v2.4.7 using an HKY plus gamma site model with a strict clock model after temporal signal was assessed using TempEst (*8*,*9*). Priors were set to the default coalescent Bayesian skyline model. The chain was run for 100 million iterations and sampled every 1000 iterations. The first 10% of runs were discarded as burn-in.

References

1. Seemann T. Prokka: rapid prokaryotic genome annotation. Bioinformatics. 2014;30:2068–9. PubMed https://doi.org/10.1093/bioinformatics/btu153

2. Bankevich A, Nurk S, Antipov D, Gurevich AA, Dvorkin M, Kulikov AS, et al. SPAdes: a new genome assembly algorithm and its applications to single-cell sequencing. J Comput Biol. 2012;19:455–77. PubMed https://doi.org/10.1089/cmb.2012.0021

3. Alikhan N-F, Petty NK, Ben Zakour NL, Beatson SA. BLAST Ring Image Generator (BRIG): simple prokaryote genome comparisons. BMC Genomics. 2011;12:402. PubMed https://doi.org/10.1186/1471-2164-12-402

4. He M, Miyajima F, Roberts P, Ellison L, Pickard DJ, Martin MJ, et al. Emergence and global spread of epidemic healthcare-associated *Clostridium difficile*. Nat Genet. 2013;45:109–13. PubMed https://doi.org/10.1038/ng.2478

5. Arndt D, Grant JR, Marcu A, Sajed T, Pon A, Liang Y, et al. PHASTER: a better, faster version of the PHAST phage search tool. Nucleic Acids Res. 2016;44:W16-21. PubMed https://doi.org/10.1093/nar/gkw387

6. Croucher NJ, Page AJ, Connor TR, Delaney AJ, Keane JA, Bentley SD, et al. Rapid phylogenetic analysis of large samples of recombinant bacterial whole genome sequences using Gubbins. Nucleic Acids Res. 2014;43:e15. https://doi.org/10.1093/nar/gku1196

7. Stamatakis A. RAxML version 8: a tool for phylogenetic analysis and post-analysis of large phylogenies. Bioinformatics. 2014;30:1312–3. PubMed https://doi.org/10.1093/bioinformatics/btu033

8. Bouckaert R, Heled J, Kühnert D, Vaughan T, Wu C-H, Xie D, et al. BEAST 2: a software platform for Bayesian evolutionary analysis. PLOS Comput Biol. 2014;10:e1003537. PubMed https://doi.org/10.1371/journal.pcbi.1003537

9. Rambaut A, Lam TT, Max Carvalho L, Pybus OG. Exploring the temporal structure of heterochronous sequences using TempEst (formerly Path-O-Gen). Virus Evol. 2016;2:vew007. PubMed https://doi.org/10.1093/ve/vew007

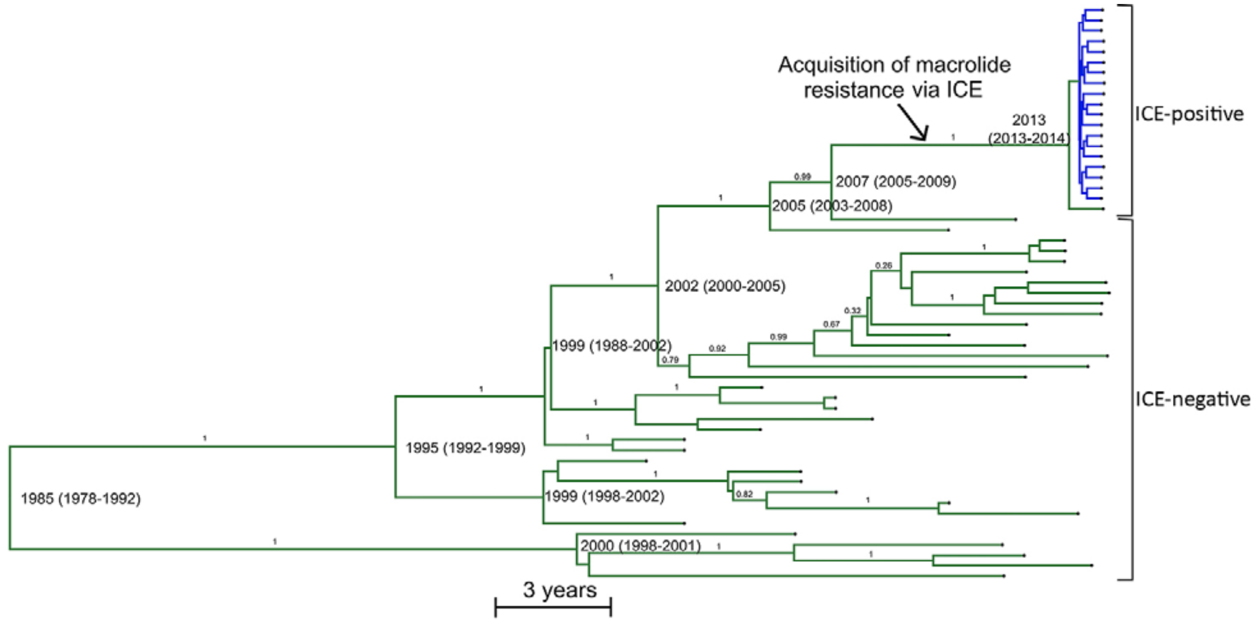

Appendix Figure. Bayesian temporal phylogenetic estimation of outbreak and non-outbreak *emm*81 *S. pyogenes* isolates from New Zealand, inferred by BEAST analysis. Node labels indicate the estimated age of the node, with 95% highest posterior densities given in parentheses. Branch labels indicate posterior values. Green branches indicate non-outbreak isolates, blue branches indicate outbreak isolates.
